# Cyclodextrins permeabilize DPPC liposome membranes: a focus on cholesterol content, cyclodextrin type, and concentration

**DOI:** 10.3762/bjoc.19.115

**Published:** 2023-10-17

**Authors:** Ghenwa Nasr, Hélène Greige-Gerges, Sophie Fourmentin, Abdelhamid Elaissari, Nathalie Khreich

**Affiliations:** 1 Bioactive Molecules Research Laboratory, Faculty of Sciences, Lebanese University, Jdeidet el-Metn 90656, Lebanon; 2 University Lyon, University Claude Bernard Lyon 1, CNRS, ISA-UMR 5280, 69622, Villeurbanne, France; 3 Unité de Chimie Environnementale et Interactions sur le Vivant (UCEIV, UR 4492), SFR Condorcet FR CNRS 3417, Université du Littoral Côte d'Opale, 145 Av. M.Schumann, 9140 Dunkirk, France

**Keywords:** cholesterol, cyclodextrins, liposome, membrane permeability

## Abstract

Cyclodextrins (CDs) are known for their ability to extract lipid components from synthetic and biological membranes and therefore to induce an increase of membrane permeability. However, the effect of cholesterol (CHOL) content in the membrane on the CD permeabilizing effect was not considered yet. Given that an increase in CHOL content reduces the membrane permeability, the aim of this work was to reveal how CHOL would modulate the CDs effect on the membrane. Hence, liposomes made of dipalmitoyl phosphatidylcholine (DPPC) and various CHOL contents (DPPC/CHOL 100:10, 100:25, 100:50, and 100:100) encapsulating the hydrophilic fluorophore, sulforhodamine B (SRB), were prepared and exposed to the native CDs (α-CD, β-CD, γ-CD) and four β-CD derivatives: the randomly methylated-β-CD (RAMEB), the low methylated-β-CD (CRYSMEB), the hydroxypropyl-β-CD (HP-β-CD) and the sulfobutyl ether-β-CD (SBE-β-CD) at different CD/DPPC molar ratios (1:1, 10:1, and 100:1). The membrane permeability was monitored following the release of SRB with time. The results demonstrated that the CDs effect on the membrane depends on the CD type, CD concentration, and membrane CHOL content. The investigated CDs exhibited an instantaneous permeabilizing effect promoting vesicle leakage of SRB from the various membranes; this effect increased with CDs concentration. Among the studied CDs, α-CD, β-CD, and RAMEB were the most permeabilizing CDs on the different membranes. Similar modifications of SRB release from the various liposomal formulations were obtained with HP-β-CD, CRYSMEB, and SBE-β-CD. γ-CD was the less potent CD in affecting the membrane permeability. The CDs effect also depended on the CHOL content: at the CD/DPPC molar ratio (100:1), RAMEB and β-CD considerably permeabilized the membrane of high CHOL content (50%, 100%) while the remaining CDs showed a decreasing permeabilizing effect upon CHOL content membrane increase.

## Introduction

Cyclodextrins (CDs) are a family of cyclic oligosaccharides made of glucopyranose units connected by α-1,4-glycosidic bonds. They possess a cone-shaped molecular structure with a hydrophobic internal cavity and a hydrophilic outer surface [1]. The common CDs are the native α-CD, β-CD, and γ-CD consisting of 6, 7, and 8 ᴅ-glucopyranose units, respectively. Due to their limited water solubility (especially β-CD), native CDs can be chemically or enzymatically modified (by e.g., alkylation, arylation, hydroxypropylation, amination, etherification, etc.) giving rise to synthetic CD derivatives with greater water solubility [2]. Thanks to their unique structure, CDs can offer exclusive advantages by allowing the entrapment of lipophilic molecules inside their inner cavities. This inclusion improves the chemical stability and aqueous solubility of the guest molecule and results in most of the cases in the formation of a water-soluble CD–guest complex [3]. Being recognized as non-toxic, biodegradable, and sustainable carriers, CDs have attracted wide interest as potential carriers in different fields, mainly in drug delivery where they are used as pharmaceutical excipients to increase the drug permeability through biological membranes improving drug bioavailability and efficacy [2,4–5]. Furthermore, the CDs peculiarities helped to develop a combined system in which CD–guest complexes are encapsulated in the aqueous core of liposomes which is generally known as “drug-in-cyclodextrin-in-liposomes” (DCL) [6]. This novel delivery system has gained popularity in the past few decades and many publications proved its importance and significance. Actually, the use of the two delivery systems (liposomes and CDs) was shown to combine the advantages of each separate system and to circumvent the drawbacks of liposomes and the problems associated with CDs: for instance, studies reported that the DCL increased the entrapment of hydrophobic drugs in liposomes and enhanced the vesicle stability. The DCL avoids a burst release of the drug from the carrier resulting in an ameliorated controlled release [6–7].

Nevertheless, CDs are known to induce considerable damages in the membrane structure and composition. In fact, CDs can alter the biophysical properties of the membrane by increasing its fluidity and permeability [8]. They are even able to extract the lipid membrane components leading the membrane to lose its integrity [8]. This behavior was attributed to the hemolytic activity of CDs previously observed on erythrocytes and other cell membranes [9].

Numerous reports highlighting the CDs-mediated lipid extraction demonstrated that some CDs displayed a higher affinity towards phospholipids such as α-CD for phosphatidylinositol (PI), phosphatidylserine (PS), and dipalmitoyl phosphatidylcholine (DPPC) [10–11], etc., while other CDs preferentially extracted cholesterol (CHOL) from membranes such as β-CD and its methylated derivatives [12–13]. Consequently, CDs are classified as permeabilizing agents for being able to promote the leakage of liposomal membranes [14]. Although a great number of reports demonstrated the membrane-damaging effect induced by several CDs, the CHOL content in the membrane was not considered in the literature despite the remarkable effect of CHOL on the stability of the lipid bilayer. In fact, CHOL can greatly modulate the membrane permeability: a previous work showed that increasing the CHOL content in the membrane results in a decrease in the membrane permeability in a dose-dependent manner [15]. Additionally, the CHOL content was demonstrated to reduce and sometimes to inhibit the permeability of DPPC vesicles induced by bioactive agents [16–17]. Given the condensing and ordering effect that CHOL exerts on the membrane, the presence of CHOL in the lipid bilayer introduced a new phase to the membrane referred to as “the liquid-ordered” (Lo) alongside with the gel phase and the liquid-disordered phase [18].

Besides, CHOL is a major component of the so-called “lipid rafts” which are perceived as membrane domains rich in CHOL and sphingomyelin and involved in various cellular processes, (e.g., signaling transduction, proteins trafficking, etc.) [19]. However, many discrepancies could be found in the literature regarding the existence of lipid rafts in synthetic membranes, especially CHOL–lipid binary mixtures. Studies of DPPC:CHOL bilayers have elucidated the formation of nanodomains enriched in CHOL within the membrane displaying a fluid-like structure as manifested in the Lo phase [20].

**Figure 1:**
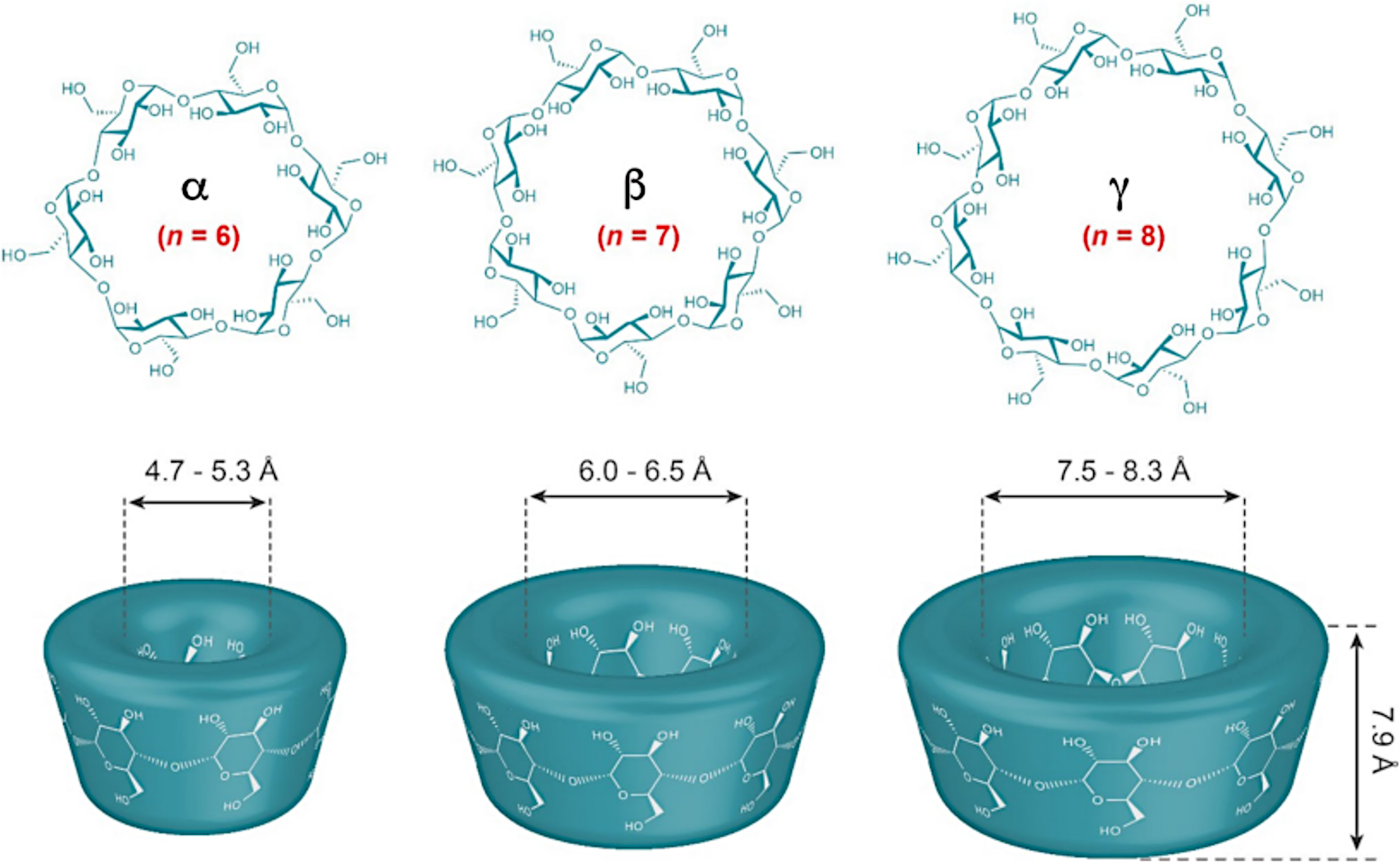
The chemical structure of the native CDs, their three-dimensional structure, and their dimensions (*n* = 6, 7, and 8 glucopyranose units for α-, β-, and γ-CD, respectively). This figure was reused by permission from Springer Nature from [21]. (“130 years of cyclodextrin discovery for health, food, agriculture, and the industry: a review” by N. Morini-Crini; S. Fourmentin; É. Fenyvesi; E. Lichtfouse; G. Torri; M. Fourmentin; G. Crini, Environmental Chemistry Letters, Vol. 19, pp 2581–2617, 2021), Copyright 2021 Springer Nature. Journal home page: https://www.springer.com/journal/10311. This content is not subject to CC BY 4.0.

**Figure 2:**
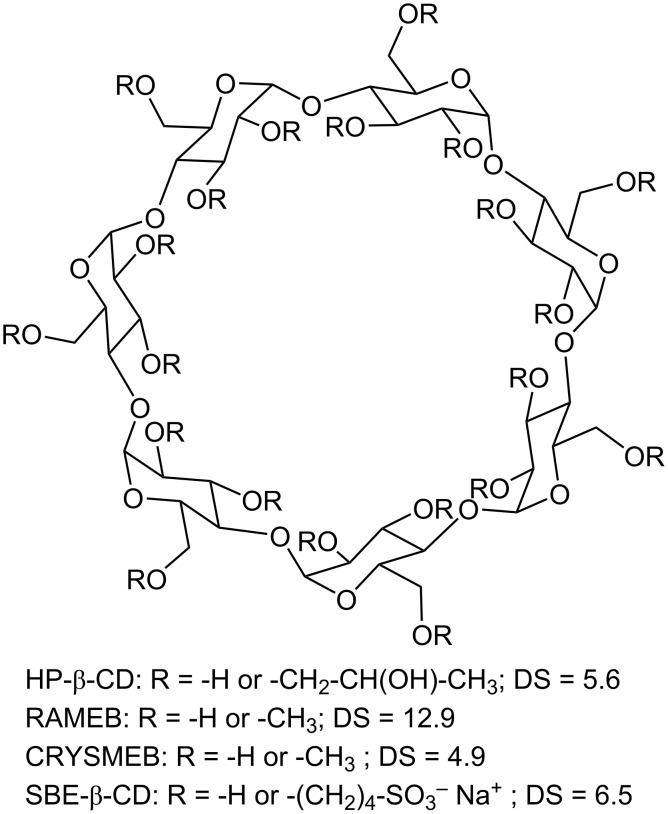
Structures of β-CD derivatives and their degrees of substitution (DS).

Different types of CDs were considered in this study; these include the native CDs: α-CD, β-CD, γ-CD, and four β-CD derivatives: the randomly methylated-β-CD (RAMEB), the low methylated-β-CD (CRYSMEB), the hydroxypropyl-β-CD (HP-β-CD), and the sulfobutyl ether-β-CD (SBE-β-CD). A schematic representation of the chemical structure of the native CDs and their dimensions is depicted in Figure 1, reprinted with permission from [21]. The structures of β-CD derivatives and their degrees of substitution are represented in Figure 2. The effect of the CDs on the membrane permeability was monitored by following the release of a hydrophilic fluorophore, sulforhodamine B (SRB), from liposomes composed of DPPC and different CHOL content upon exposure to different concentrations of CDs.

To the best of our knowledge, this work is the first study investigating the effect of CDs on the permeability of DPPC liposome membranes of various CHOL content. The CDs effect was examined at various CD/DPPC molar ratios. This work will provide a better understanding of the influence of CHOL content on the CDs effect with regards to their affinity to lipid membrane components. It will also allow us to point out if the CDs-induced lipid extraction may occur in “lipid rafts”.

## Results and Discussion

**Figure 3:**
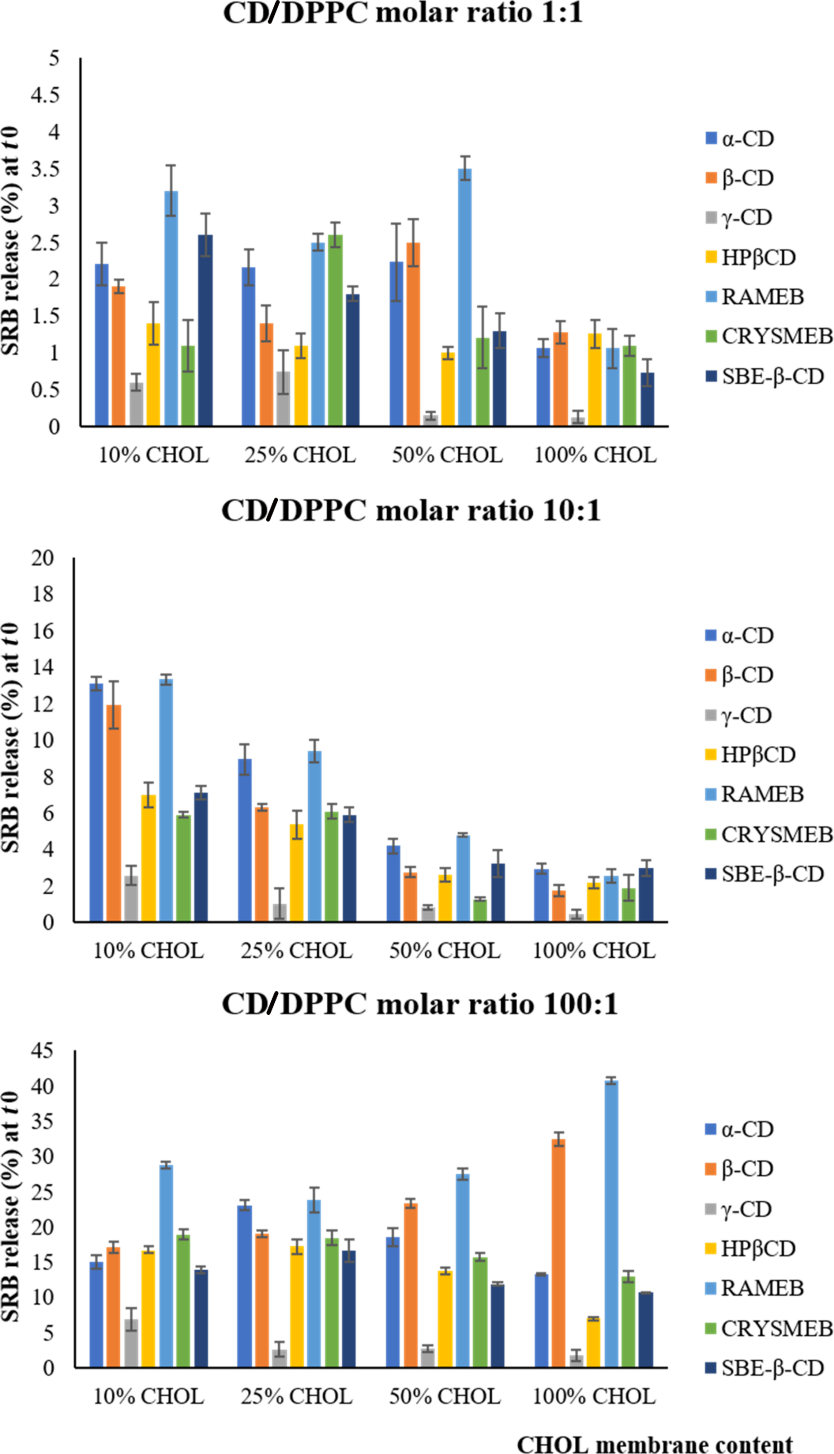
The instantaneous effect of CDs on the various liposome membranes at different CD/DPPC molar ratios (1:1, 10:1, and 100:1) at *t*_0_. Values are expressed as the means of three different measurements ± SD.

**Figure 4:**
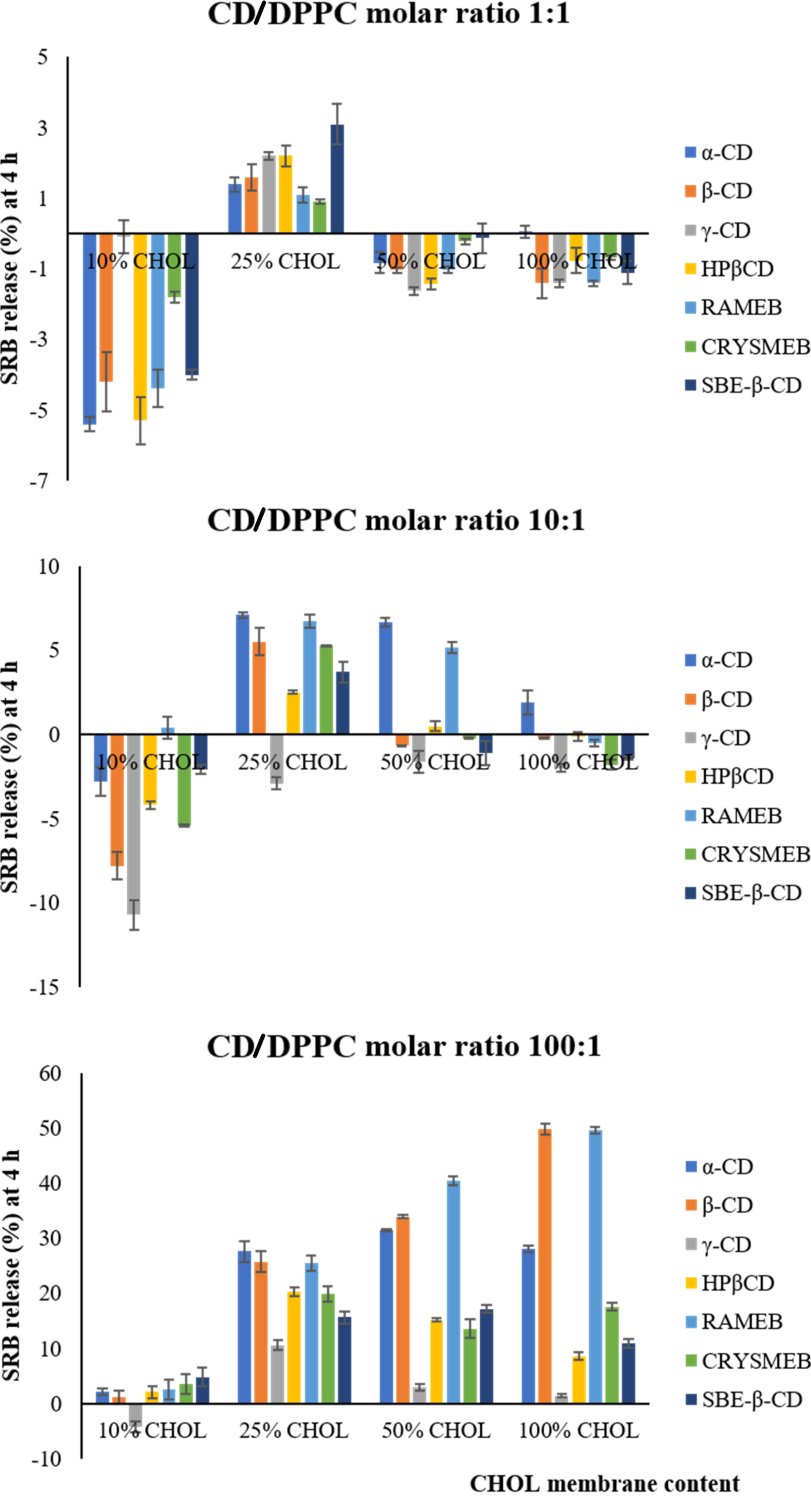
The permeabilizing effect of CDs on the various liposome membranes at different CD/DPPC molar ratios (1:1, 10:1, and 100:1) obtained at 4 h. Values are expressed as the means of three different measurements ± SD.

**Figure 5:**
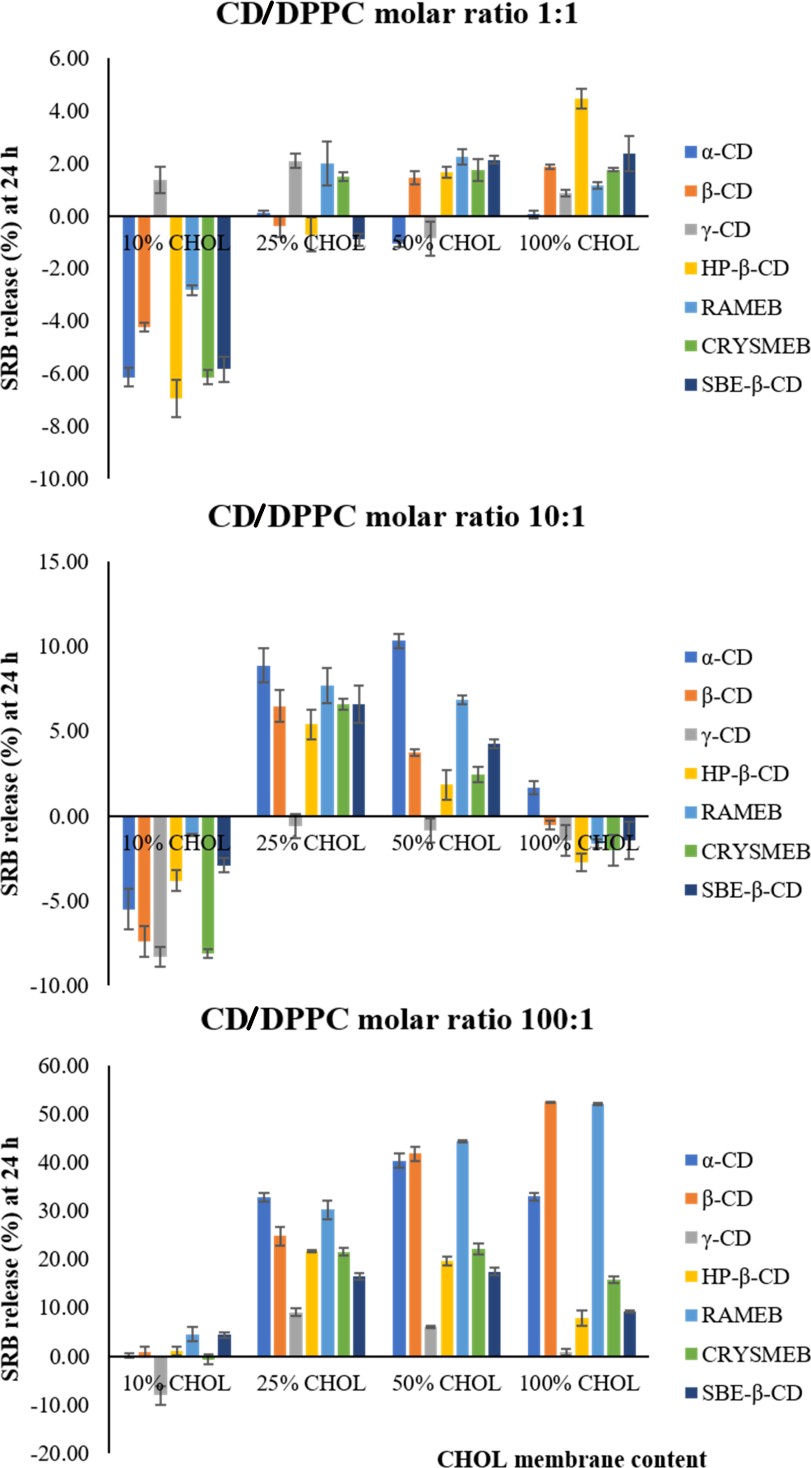
The permeabilizing effect of CDs on the various liposome membranes at different CD/DPPC molar ratios (1:1, 10:1, and 100:1) obtained at 24 h. Values are expressed as the means of three different measurements ± SD.

In this study, liposome membranes made of DPPC and various CHOL contents were prepared (10%, 25%, 50%, and 100% CHOL). It is relevant to note that the percentage of CHOL in the formulations represents the amount of CHOL added to the fixed amount of DPPC not the sum of the total lipids. Thus, a formulation of 100% CHOL comprises a number of moles of CHOL equal to that of DPPC. These formulations were individually treated with 0.15 mM, 1.5 mM, and 15 mM of CDs (CD/DPPC molar ratios 1:1, 10:1 and 100:1, respectively). Following the exposure of liposomes to CDs, the samples were incubated at 37 °C and the fluorescence signals were measured at time 0, 4, and 24 h. For each formulation, the effect of CDs was obtained by subtracting the SRB release from vesicles in the presence of CDs from that obtained in their absence as explained earlier. Results are presented in Figures 3, 4, and 5.

The obtained data are also reported in Tables S1–S4 (Supporting Information File 1) in which the vertical reading points out the effect of the CD concentration on the SRB release for a specific membrane composition, while the horizontal reading of the tables highlights the impact of CHOL content on the permeability of membranes at various intervals of time.

The SRB release kinetics for blank liposomes (untreated with CDs) obtained in our study were consistent with a previous work conducted by Kaddah et al. [15]. Indeed, the study focused on following the SRB release from liposomes composed of DPPC and various CHOL contents. The previous results showed that the SRB release from liposomes incorporating 10% CHOL was 6.1% after 1 h of incubation whereas those from membranes containing higher CHOL contents did not exceed 5% after the same time. After 4 h of incubation, the SRB release reached 16.41% for 10% CHOL liposomal membranes and less than 10% for vesicles composed of higher CHOL content. After 48 h of incubation, 63% of SRB was released from 10% CHOL membranes while less than 20% of SRB leakage was obtained with the formulations of 50 and 100% CHOL [15]. Similar findings were noted in this work (Tables S1–S4 in Supporting Information File 1) showing that increasing the CHOL content in the membrane reduces its permeability and increases its rigidity and stability.

### 1 The instantaneous effect of CDs at *t*_0_

**CD/DPPC molar ratio (1:1).** As shown in Figure 3, the studied CDs barely modified the membrane permeability of the different liposome membranes where the percentage of SRB release did not exceed 4% when compared to the blank of each formulation.

**CD/DPPC molar ratio (10:1).** α-CD, β-CD, and RAMEB were the most effective CDs inducing an increase in the permeability of the different membranes. Their maximum effect reaching 15% of SRB release was observed at the lowest CHOL content and their permeabilizing effect decreased with CHOL content increase (13.08, 8.94, 4.20, and 2.95% of SRB release from α-CD-treated liposomes composed of 10, 25, 50, and 100% CHOL, respectively).

HP-β-CD, CRYSMEB, and SBE-β-CD were less effective than α-CD, β-CD, and RAMEB on the membranes since the instantaneous SRB release values did not exceed 7% regardless the membrane composition at *t*_0_. Their highest effect was observed at a low CHOL content (7.11% of SRB release from SBE–β-CD-treated liposomes composed of 10% CHOL) with a noticeable decrease (SRB release less than 4%) with membranes of high CHOL content. γ-CD demonstrated the weakest effect on the membrane regardless its composition.

**CD/DPPC molar ratio (100:1).** At this molar ratio, RAMEB was the most effective CD acting on both CHOL-poor and -rich membranes. Remarkably, the SRB release values reached around 42.00% with membranes containing 100% CHOL which draws attention to the ability of RAMEB to extract CHOL from membranes rich in CHOL. β-CD showed a similar effect at 10% CHOL but it remained lower than that obtained with RAMEB.

Although α-CD and β-CD demonstrated the same ability to affect the permeability of membranes of CHOL content 10, 25, and 50% (with SRB release values ranging from 15 to 25%), their effect was not the same at 100% CHOL where β-CD (SRB release of 32.37%) was more potent than α-CD (SRB release of 13.29%). HP-β-CD, CRYSMEB, and SBE-β-CD similarly affected the liposome membranes of different CHOL content; their permeabilizing effect was higher on low CHOL content membranes 10 and 25% (SRB release values varying between 14 and 20%) compared to high CHOL membranes 50 and 100% (SRB release values ranging from 7 to 14%). Among these CDs, HP-β-CD exerted the lowest effect at 100% CHOL (6.9% of SRB release). As for γ-CD, it increased the SRB release from 10% CHOL membranes at this high CD concentration, though, its effect decreased with the other membrane types. Overall, we observed that increasing the CDs concentration increased their permeabilizing effects regardless the membrane composition and the CD type.

### 2 The permeabilizing effect of CDs at 4 h

**CD/DPPC molar ratio (1:1).** As depicted in Figure 4, the effect of CDs at the molar ratio 1:1 on the various membrane types did not strongly differ from the blank or untreated liposomes; less than 4% of SRB release (compared to blank) was obtained with the different CDs. Surprisingly, all CDs seem to produce a slight decrease of membrane permeability (less than 6%) when compared to the blank for the membranes of CHOL content 10, 50, and 100% whereas a slight increase (3% of release) was noticed after CDs exposure to 25% CHOL membranes.

**CD/DPPC molar ratio (10:1).** The same trends obtained at the CD/DPPC molar ratio 1:1 seem to be maintained at the CD/DPPC molar ratio 10:1. α-CD and RAMEB were the only CDs that induce an increase in SRB release higher than 5% with the membranes containing 25 and 50% CHOL. β-CD kept a weak permeabilizing effect on 25% CHOL membrane.

**CD/DPPC molar ratio (100:1).** α-CD, β-CD, and RAMEB produced a permeabilizing effect on the membranes composed of 25, 50 and 100% CHOL where the SRB release values varied between 25 and 50%. The effect of β-CD and RAMEB increased with CHOL content; this was not obtained with α-CD. HP-β-CD, CRYSMEB, and SBE-β-CD increased the permeability of 25, 50, and 100% CHOL membranes (with SRB release values 10 to 20%) with a better effect at 25 and 50% CHOL.

### 3 The permeabilizing effect of CDs at 24 h

**CD/DPPC molar ratio (1:1).** As we can see in Figure 5, the data obtained at 24 h are similar to those collected at 4 h. Effectively, at the CD/DPPC molar ratio (1:1), the investigated CDs did not exhibit a permeabilizing effect on 10% CHOL membranes. Even though some CDs showed an effect at 25, 50, and 100% CHOL, their effect remained weak and unsignificant (SRB release values less than 4% compared to blank).

**CD/DPPC molar ratio (10:1).** At the CD/DPPC molar ratio (10:1), α-CD and RAMEB were able to permeabilize 25 and 50% CHOL membranes. β-CD exerted a slight effect when compared to that of α-CD and RAMEB.

**CD/DPPC molar ratio (100:1).** A strong permeabilizing effect was induced by α-CD, β-CD, and RAMEB with SRB release values ranging from 25 to 55%. The remaining β-CD derivatives showed a lower effect at 25 and 50% CHOL.

## Discussion

The CD–membrane interaction was broadly studied in the past few decades. The ability of CDs to induce membrane permeabilization was also proved in numerous reports. This effect was dose dependent which is consistent with the obtained results where the permeabilizing effect of CDs increased with CDs concentration: at the CD/DPPC molar ratio (1:1), the CDs did not promote considerable vesicle leakage whereas at the CD/DPPC molar ratio (10:1), the CDs affected the membrane permeability and their effect was enhanced at the CD/DPPC molar ratio (100:1).

In addition, we can notice that the CDs potency in permeabilizing the membrane was not the same: among the studied CDs, and α-CD, β-CD, and RAMEB were the most potent CDs acting on both low and high CHOL content membranes, particularly at the highest CDs concentration. However, they did not behave similarly at high CHOL content: the permeabilizing effect of β-CD and RAMEB was enhanced at 100% CHOL compared to α-CD whose release effect was reduced at high CHOL content. Additionally, α-CD presented the highest effect at 25% CHOL at the highest CD concentration. The main reason behind these observations is the preferential lipid membrane extraction exhibited by the CDs. As a matter of fact, α-CD can remove phospholipids from membranes. A previous work reported that α-CD can extract DPPC through a special complex formation between the acyl chains of the phospholipid and the CD molecules where many α-CD units string along the nonpolar chains of DPPC forming a rotaxane-like ring [11]. DSC studies have also proved that α-CD forms an insoluble complex with DPPC [22]. Besides, α-CD has previously demonstrated the strongest destabilizing effect on the DPPC liposome membranes among the native CDs [11]. In our study, being more abundant in phospholipids than CHOL, the 10 and 25% CHOL liposome membranes were more sensitive to α-CD (at a CD/DPPC molar ratios above 10:1) compared to the remaining formulations (50 and 100% CHOL) which explains the highest permeabilizing effect instantly exerted by α-CD at low CHOL content. With increasing CHOL content (at 50 and 100% CHOL), CHOL might be clustered into microdomains which may hinder the α-CD’s effect and reduce its effect on the membrane.

According to literature, the effect of β-CD and RAMEB is mainly attributed to the ability of these CDs to remove CHOL from membranes [8]. This explains the increase of their permeabilizing effect with CHOL addition at the CD/DPPC molar ratio 100:1 as more CHOL molecules are extracted from the membrane leading to the disruption of the membrane continuity and its subsequent leakage [14]. Though, this effect was not seen at the CD/DPPC molar ratio of (10:1). This leads to suppose that the CHOL extraction mediated by β-CD is probably achieved at a CD/DPPC molar ratio larger than 10:1. Comparing the α-CD-induced permeability to that of β-CD and RAMEB, it is possible to state that CHOL-rich membranes are sensitive to β-CD and RAMEB at the highest ratio (100:1). Hence, β-CD and RAMEB are active on CHOL-rich membranes (50 and 100% CHOL) where the raft domains may be present. We suggest therefore that β-CD and RAMEB at high concentration would extract CHOL from raft domains, as previously reported for RAMEB [8,23].

As mentioned earlier, HP-β-CD, CRYSMEB, and SBE-β-CD were less effective on the membrane than α-CD, β-CD, and RAMEB. They displayed a better permeabilizing effect at low CHOL content (10 and 25% CHOL membranes) with a decrease in their effect upon CHOL content increase at the CD/DPPC molar ratios 10:1 and 100:1.

Despite that CRYSMEB is a methylated β-CD derivative, its behavior was different from that of RAMEB whose effects were enhanced with CHOL increase at the CD/DPPC molar ratio 100:1. Our result for CRYSMEB is in agreement with the work of Piel et al., who showed that CRYSMEB is less potent than RAMEB and other methylated CDs in promoting calcein leakage from liposomes comprising 30% CHOL [13]. The authors stated that the low degree of substitution of CRYSMEB decreased its affinity to the lipid membrane components and resulted in a weaker disturbing effect compared to RAMEB and other methylated CDs. Consequently, CRYSMEB is active on CHOL-poor membranes (25% CHOL) at a CD/DPPC molar ratio above 10:1.

HP-β-CD and SBE-β-CD showed similar behavior to CRYSMEB. According to a recent biophysical study, HP-β-CD demonstrated an increase in the fluidity of DPPC liposomes through the interaction of HP-β-CD with the polar head group region and the acyl chains of DPPC [24]. Although the complex between HP-β-CD and CHOL has been previously characterized [25], this complex seems to be unstable. Thus, a better interaction would occur between HP-β-CD and DPPC rather than with CHOL which explains the obtained results. As for SBE-β-CD, it was reported that charged CDs could not interact with CHOL molecules and form inclusion complexes due to charge repulsion [26]. This could explain the results obtained for these two CDs. Hence, HP-β-CD and SBE-β-CD are active on CHOL-poor membranes.

The effects of β-CD derivatives obtained in this study present a good correlation with biological membranes studies: the methylated β-CD derivatives with high degree of substitution (RAMEB in our study) possess the strongest CHOL extraction capacity and can subsequently achieve the highest solubilization of CHOL [27], while the low-substituted derivatives (CRYSMEB in our case) were less cytotoxic and maintained the integrity of endothelial cells assuming a lower affinity to CHOL membrane compared to the other derivatives [23]. Furthermore, the hydroxypropyl substituents are bulkier and less hydrophobic than the methyl groups resulting in a lower CHOL solubilizing capacity and a weaker hemolytic activity for HP-β-CD [28]. Besides, the ionic β-CD derivatives are less effective in promoting CHOL extraction given that the charge decreases the affinity of CDs towards CHOL [27].

With regards to the native γ-CD, it exerted the weakest effect among all CDs. A slight permeabilizing effect was instantly obtained on the various membranes at the highest CD concentration and it disappeared with time. These observations are in agreement with published data where γ-CD always exhibited low vesicle leakage [29]. Actually, γ-CD was found to be less lipid specific than the remaining native CDs (α-CD and β-CD) [8], which implies that the interaction of γ-CD with DPPC would be not favorable. Considering its large cavity in comparison to α and β-CD, γ-CD is not able to extract properly lipid membrane components. This result confirms that the mechanism of CDs-induced permeability is mainly attributed to the lipid extraction mediated by CDs resulting in the formation of a complex between the CD and the lipid membrane components, as reviewed by Nasr et al. in 2020 [14]. Based on our results, γ-CD was active on CHOL-poor membranes. Yet, its effect remains very weak compared to the studied CDs.

Moreover, the instantaneous permeabilizing effect of CDs on 10% CHOL membranes disappeared at 4 and 24 h regardless the CD type and the CD/DPPC molar ratio (Supporting Information File 1). The instantaneous effect of CDs on other membranes was similarly obtained. This may be due to the rapid equilibrium that could be established at 10% CHOL between the membrane and the CD. In fact, CD can instantly interact with the liposome membrane constituents and the extraction of lipid molecules takes place resulting in membrane destabilization. This is illustrated by the rapid leakage of SRB loaded liposomes upon CDs exposure to membranes of different CHOL contents. After this initial effect, the CD would not influence the stability of the bilayer supposing that the membrane is re-organized and the equilibrium between the CD and the membrane is established. Our result for 10% CHOL liposomes is in accordance with that of Hatzi et al. showing an instant calcein release from CHOL-free liposomes (PC and H-PC vesicles) exposed to CDs with no further leakage with time [12]. It is worthy to note, that the 10% CHOL membranes are less stable than the remaining formulations and evidenced the same SRB release pattern as CHOL-free liposomes [15]. Nonetheless, the rapid equilibrium between the CDs and the membrane cannot alone explain the reason behind the disappearance of the CDs effect with time because it does not fully consider the complexation process between the CDs and the membrane components. Another finding obtained by Nishijo and his co-workers [30] may further clarify this idea. The authors studied the interaction of various CDs with CHOL: heptakis(2,6-di-*O*-methyl)-β-CD (DOM-β-CD) was able to form two types of soluble complexes, with molar ratios of 1:1 and 1:2 (CHOL/DOM-β-CD). The latter (1:2 inclusion complex) occurred much more easily than that of the 1:1 complex showing a much higher equilibrium constant. At low CDs concentration, the formation of the 1:1 inclusion complex dominated with low equilibrium constant (109 M^−1^) suggesting that the unstable complex would rapidly decompose into its components. With time elapsing and with increasing CDs concentration, the 1:1 inclusion complex was transformed into the more stable 1:2 complex with greater equilibrium constant (5.68 × 10^4^ M^−1^). Therefore, we can suggest that at high CDs concentration, more of the lipid membrane components would enter the cavity of CDs to form a stable complex instead of refluxing back to the liposomes. Based on these studies, we can assume that the disappearance of the CDs permeabilizing effect with time is additionally accounted for the complexation process occurring between the CDs and the membrane components [30].

Interestingly, a decrease in the permeability was reported with various CDs after *t*_0_ (Figure 2 and Figure 3). This result could be in line with the ability of CDs to stabilize the biological membranes during freeze-drying [8].

Considering the above discussed results, we can assume the dependency of the CDs effect on the membrane permeability on three main parameters: the CHOL content, the CD concentration, and type or more precisely its affinity towards lipid membrane components. At the CD/DPPC molar ratio 1:1, the studied CDs had no effect on the membrane regardless the CHOL content. Their effect occurred above this ratio and was thereafter strongly modulated by the CHOL content depending on the CD’s affinity or interaction with lipid membrane components. CHOL-poor membranes were mainly sensitive to the CDs displaying a preferential phospholipids membrane extraction such as α-CD and the β-CD derivatives: HP-β-CD, CRYSMEB, and SBE-β-CD with α-CD being the most potent, whereas CHOL-rich membranes were sensitive to β-CD and its methylated derivative, RAMEB.

## Conclusion

In this work, we investigated the effect of CDs on the membrane permeability of DPPC liposomes composed of different CHOL contents at different CD/DPPC molar ratios. The obtained data revealed the dependency of the CD’s induced permeability on three main factors: the CHOL content, the CD concentration, and the CD type interpreted by their ability to extract lipid membrane components. No effect was observed for the CD/DPPC molar ratio 1:1 on the membrane permeability for all the CDs. At the ratio 10:1 and 100:1, CDs exhibited different behaviors towards the membrane depending on the CHOL content and the CDs’ affinity to the lipid membrane components. Among the studied CDs, α-CD, β-CD, and RAMEB can be classified as the most effective CDs acting on both CHOL-rich and -poor membranes with β-CD and RAMEB showing an enhanced effect at high CHOL content. Hence, β-CD and RAMEB may extract CHOL from raft domains at high CHOL content. The remaining β-CD derivatives (HP-β-CD, CRYSMEB, and SBE-β-CD) showed a lower effect that was mainly observed instantaneously at low CHOL content and it decreased with CHOL content increase. γ-CD showed the weakest effect on the membrane. Increasing time of incubation did not affect the CD permeabilizing effect on the various liposomal membranes.

These results contribute to the better understanding of the CD–membrane interaction and may be very useful in the choice of these CDs as a delivery system. Furthermore, these results may help in the development of the combined delivery system “drug-in-cyclodextrin-in-liposomes” (DCL) where CD–drug inclusion complexes are in contact with the membrane. The choice of CD in such a system does not only depend on the drug affinity towards the CD cavity, but should also take into consideration the affinity of the selected CD towards membrane lipids, the CD–phospholipid molar ratio, and the CHOL content in the membrane.

## Experimental

### Materials and methods

#### Materials

α-CD, β-CD, γ-CD, and randomly methylated-β-CD (RAMEB, DS = 12.9), were provided by Wacker Chemie (Germany). Low methylated-β-CD (CRYSMEB, DS = 4.9) and hydroxypropyl-β-CD (HPBCD, DS = 5.6) were provided by Roquette Frères (Lestrem, France). Sulfobutyl ether-β-CD (SBE-β-CD, DS = 6.5) was provided by LIGAND Pharmaceuticals (San Diego, CA, USA). Dipalmitoylphosphatidylcholine (DPPC) and trizma base (buffer reagent) were purchased from Sigma-Aldrich, Switzerland. Triton X-100, sodium chloride (NaCl), and Sephadex G25 gel were purchased from Sigma-Aldrich, Belgium. Ammonium molybdate, hydrogen peroxide, potassium dihydrogen phosphate, sodium sulfite, sodium bisulfite, chloroform, and methanol were purchased from Sigma-Aldrich, Germany. Cholesterol and sulforhodamine B were purchased from Sigma-Aldrich, USA. 4-Amino-3-hydroxy-1-naphthalene sulfonic acid was purchased from Fluka, India. Sulfuric acid was purchased from ACROS Organics, Belgium and diethyl ether was purchased from VWR-Prolabo Chemicals, Belgium.

#### Liposomes preparation, extrusion, and purification

The SRB-loaded liposomes were prepared, extruded, and purified following the same method described by Nasr et al. [17]. Briefly, the lipid mixture of DPPC and CHOL at the different molar ratios (DPPC/CHOL 100:10, 100:25, 100:50, and 100:100) was dissolved in an organic phase made of chloroform/diethyl ether/methanol 6:6:1 (v/v/v). After a short sonication, the aqueous phase made of SRB (150 mM) dissolved in Tris HCl buffer (0.1 M, pH 7.4) was added to the lipid solution and the mixture was sonicated at 60 °C under a nitrogen stream. The removal of organic solvents was achieved by evaporation at 45 °C using a rotary vacuum evaporator (Heidolph, Germany). Then, the aqueous phase (SRB containing buffer) was added to the dry film and the mixture was sonicated at 60 °C under a nitrogen stream to generate vesicles. The SRB-loaded liposomes were subjected to extrusion through polycarbonate filter membranes (Avanti Polar Lipids, Switzerland) of decreasing pore sizes resulting in a homogenous mixture of LUVs (large unilamellar vesicles).

Finally, the purification of the SRB-loaded LUVs to eliminate unencapsulated SRB and lipid molecules from liposomes was carried out via a centrifugation (2 hours, 15 000 rpm, 4 °C) and a molecular sieves chromatography (using a Sephadex G25 gel filtration column). A Tris HCl buffer (0.1 M, pH 7.4) containing 150 mM NaCl was used for elution and liposome storage.

#### Exposure of SRB-loaded liposomes to CDs

The concentration of DPPC was determined for each formulation according to Bartlett method, as previously described by Habib et al. [31]. Then, the liposomal suspensions were all diluted to obtain solutions with a DPPC concentration of 0.15 mM. At time of incubation, the CDs were individually added to the liposomes so that the concentration of CD in the final volume of CD treated liposomes is equal to 0.15 mM, 1.5 mM, and 15 mM in respect to the CD/DPPC molar ratios: 1:1, 10:1, and 100:1, respectively. The fluorescence signal was measured for each sample immediately after the exposure of LUVs to CDs and the samples were incubated at 37 °C during 24 h. For each formulation, a solution containing only liposomes was used as the blank solution.

#### The membrane permeability study by fluorescence spectroscopy

The membrane permeability is commonly evaluated by following the leakage of self-quenching probes such as SRB from liposomes [14]. Indeed, a fluorescence auto-quenching effect is observed when SRB is encapsulated at a high concentration inside the liposomes. The recovery of the fluorescence signal is achieved upon the release of the dye from liposomes and its dilution in the external medium. Thus, the effect of CDs on the permeability of liposomal membranes was studied by measuring the fluorescence signal of liposomes treated with CDs and incubated at 37 °C. An enhanced membrane permeability is detected when the fluorescence signal is increased demonstrating the permeabilizing effect of the tested agents.

As described in the previous section, the SRB-loaded liposomes of each formulation treated or not with CDs were incubated in a water bath at 37 °C. Aliquots were taken from each sample at 0, 4, and 24 h and the fluorescence signal was measured after a dilution of 100 times in the Tris HCl (0.1 M, pH 7.4) buffer containing 150 mM NaCl.

The measurements were carried out on a spectrophotometer (Hitashi F-7000 Spectrofluorometer) at an excitation wavelength of 535 nm and an emission wavelength of 590 nm. The emission spectrum was recorded in the range 540–700 nm. The results of the permeability study were expressed as the percentage of the fluorophore released from LUVs obtained using Equation 1:


[1] $$\text{SRB release percentage =}\frac{\left(F_{t}-F_{0,\text{blank}}\right)}{F_{\max}}\times 100,$$

where *F*_t_ is the fluorescence intensity measured at time *t* for each sample, *F*_0,blank_ is the fluorescence intensity measured at time 0 for the blank liposomes of each formulation and *F*_max_ is the maximum fluorescence indicating a complete release of SRB from vesicles and obtained in the presence of the nonionic detergent, Triton X-100 (1%) in a Tris HCl buffer (0.1 M, pH 7.4) containing 150 mM NaCl. The results are expressed as the means of three independent experiments ± SD.

To highlight the effect of CDs on the membrane, results are presented by subtracting the SRB release obtained in the presence of CDs from that obtained in their absence.

#### Statistical analysis

To assess significant differences between values, statistical analysis was carried out using the Student’s t-test. A value of *p* < 0.05 was considered statistically significant.

## Supporting Information

File 1 Supporting tables.
