# Supplementary material for: Cyclodextrins permeabilize DPPC liposome membranes: a focus on cholesterol content, cyclodextrin type, and concentration
Source: Beilstein J Org Chem. 2023 Oct 17;19:1570–9. doi: 10.3762/bjoc.19.115 (PMC10616703; doi:10.3762/bjoc.19.115)
Supplement: File 1 — Supporting tables. [file Beilstein_J_Org_Chem-19-1570-s001.pdf]

BEILSTEIN JOURNAL OF ORGANIC CHEMISTRY

Supporting Information

for

Cyclodextrins permeabilize DPPC liposome membranes: a focus on cholesterol content, cyclodextrin type, and concentration

Ghenwa Nasr, Hélène Greige-Gerges, Sophie Fourmentin, Abdelhamid Elaissari and Nathalie Khreich

Beilstein J. Org. Chem. **2023,** *19,* 1570–1579. doi:10.3762/bjoc.19.115

Supporting tables

License and Terms: This is a supporting information file under the terms of the Creative Commons Attribution License (https://creativecommons.org/licenses/by/4.0). Please note that the reuse, redistribution and reproduction in particular requires that the author(s) and source are credited and that individual graphics may be subject to special legal provisions.
The license is subject to the *Beilstein Journal of Organic Chemistry* terms and conditions: (https://www.beilstein-journals.org/bjoc/terms)

Table S1: The SRB release values obtained at t_0, 4 h and 24 h for α-CD-treated liposomes of different CHOL contents and different CD/DPPC molar ratios.

The SRB release value of blank liposomes was 0% at t_0 for the different formulations. Values are expressed as the means of three different measurements ± SD (* indicates a value of $p < 0.05$ statistically significant. The p values compare the membrane permeability in the presence of CD to that of the respective blank).

	**DPPC/CHOL (100:10)**			**DPPC/CHOL (100:25)**			**DPPC/CHOL (100:50)**			**DPPC/CHOL (100:100)**		
**Time**	**0 h**	**4 h**	**24 h**	**0 h**	**4 h**	**24 h**	**0 h**	**4 h**	**24 h**	**0 h**	**4 h**	**24 h**
**Blank**	0	43.64 ± 1.84	48.34 ± 1.73	0	12.60 ± 1.28	15.01 ± 1.35	0	3.13 ± 0.79	4.17 ± 1.26	0	2.41 ± 0.70	5.15 ± 1.35
**α-CD/DPPC 1:1**	2.20 ± 0.39*	38.20 ± 0.97*	42.19 ± 0.35*	2.16 ± 0.42*	13.99 ± 0.72	15.13 ± 0.40	2.23 ± 0.53*	2.31 ± 0.30	3.14 ± 0.13	1.06 ± 0.12	2.46 ± 0.86	5.20 ± 0.34
**α-CD/DPPC 10:1**	13.08 ± 0.38*	40.84 ± 0.86*	42.76 ± 1.20*	8.94 ± 0.82*	19.67 ± 0.17*	23.85 ± 1.01*	4.20 ± 0.39*	9.81 ± 0.27*	14.48 ± 0.43*	2.95 ± 0.25*	4.27 ± 0.73*	6.85 ± 0.39*
**α-CD/DPPC 100:1**	15.00 ± 0.95*	45.70 ± 1.63	48.38 ± 1.87	23.01 ± 0.73*	40.16 ± 1.85*	47.80 ± 0.85*	18.50 ± 1.23*	34.55 ± 0.18*	44.48 ± 1.45*	13.29 ± 0.12*	30.50 ± 0.54*	38.06 ± 0.74*

Table S2: The SRB release values obtained at time t_0, 4, and 24 h for β-CD-treated liposomes at different CHOL content and different CD/DPPC molar ratios.

The SRB release value of blank liposomes was 0% at t_0 for the different formulations. Values are expressed as the means of three different measurements ± SD (* indicates a value of $p < 0.05$ statistically significant. The p values compare the membrane permeability in the presence of CD to that of the respective blank.)

	**DPPC/CHOL (100:10)**			**DPPC/CHOL (100:25)**			**DPPC/CHOL (100:50)**			**DPPC/CHOL (100:100)**		
**Time**	**0 h**	**4 h**	**24 h**	**0 h**	**4 h**	**24 h**	**0 h**	**4 h**	**24 h**	**0 h**	**4 h**	**24 h**
**Blank**	0	43.64 ± 1.84	48.34 ± 1.73	0	12.60 ± 1.28	15.01 ± 1.35	0	3.13 ± 0.79	4.17 ± 1.26	0	2.41 ± 0.70	5.15 ± 1.35
**β-CD/DPPC 1:1**	1.90 ± 0.09*	39.45 ± 1.03	44.05 ± 0.16	1.40 ± 0.25	14.24 ± 0.37	14.60 ± 0.59	2.50 ± 0.32*	2.14 ± 0.08	5.63 ± 0.25	1.28 ± 0.15	0.99 ± 0.43	7.02 ± 0.08
**β-CD/DPPC 10:1**	11.91 ± 1.28*	35.79 ± 0.81*	40.85 ± 0.90*	6.32 ± 0.20*	18.07 ± 0.82*	21.45 ± 0.94*	2.76 ± 0.28*	2.53 ± 0.01	7.93 ± 0.20*	1.75 ± 0.30*	2.16 ± 0.01	4.60 ± 0.24
**β-CD/DPPC 100:1**	17.10 ± 0.83*	44.71 ±1.23	49.20 ± 2.02	18.94 ± 1.48*	38.29 ± 1.89*	39.78 ± 1.95*	23.30 ± 0.63*	36.97 ± 0.33*	45.85 ± 1.54*	32.37 ± 0.90*	52.3 ± 1.00*	57.71 ± 0.01*

Table S3: The SRB release values obtained at t_0, 4, and 24 h for the β-CD derivatives-treated liposomes of different CHOL content and at different CD/DPPC molar ratios.

The SRB release value of blank liposomes was 0% at t_0 for the different formulations. Values are expressed as the means of three different measurements ± SD (* indicates a value of $p < 0.05$ statistically significant. The p values compare the membrane permeability in the presence of CD to that of the respective blank).

	DPPC/CHOL (100:10)			DPPC/CHOL (100:25)			DPPC/CHOL (100:50)			DPPC/CHOL (100:100)		
**Time**	**0 h**	**4 h**	**24 h**	**0 h**	**4 h**	**24 h**	**0 h**	**4 h**	**24 h**	**0 h**	**4 h**	**24 h**
**Blank**	0	43.64 ± 1.84	48.34 ± 1.73	0	12.60 ± 1.28	15.01 ± 1.35	0	3.13 ± 0.79	4.17 ± 1.26	0	2.41 ± 0.70	5.15 ± 1.35
**RAMEB**												
**CD/DPPC 1:1**	3.20 ± 0.44*	39.22 ± 0.53	45.49 ± 0.19	2.46 ± 0.11*	13.73 ± 0.22	16.97 ± 0.83	3.50 ± 0.16*	2.09 ± 0.09	6.38 ± 0.30	1.06 ± 0.27	0.97 ± 0.18	6.34 ± 0.11
**CD/DPPC 10:1**	13.32 ± 0.27*	43.99 ± 0.65	47.25 ± 0.06	9.39 ± 0.64*	19.32 ± 0.42*	22.72 ± 1.02*	4.78 ± 0.09*	8.35 ± 0.33*	11.04 ± 0.27*	2.56 ± 0.37*	1.90 ± 0.21	3.47 ± 0.28
**CD/DPPC 100:1**	28.70 ± 0.48*	46.06 ± 1.75	52.76 ± 1.51*	23.80 ± 1.77*	38.13 ± 1.40*	45.24 ± 1.87*	27.50 ± 0.82*	43.51 ± 0.80*	48.46 ± 0.23*	40.74 ± 0.46*	52.00 ± 0.52*	57.07 ± 0.24*
**CRYSMEB**												
**CD/DPPC 1:1**	1.11 ± 0.53	41.81 ± 0.16	42.24 ± 0.28	2.57 ± 0.17*	13.53 ± 0.06	16.45 ± 0.17	1.21 ± 0.42	2.85 ± 0.78	5.89 ± 0.40	1.09 ± 0.14	1.72 ± 0.71	6.89 ± 0.07
**CD/DPPC 10:1**	5.90 ± 0.14*	38.18 ± 0.06	40.21 ± 0.27	6.09 ± 0.38*	17.87 ± 0.01	21.64 ± 0.31*	1.28 ± 0.10*	2.92 ± 0.05	6.64 ± 0.48*	1.85 ± 0.72*	0.59 ± 0.30	3.12 ± 0.93
**CD/DPPC 100:1**	18.90 ± 0.68*	47.07 ± 1.72	47.55 ± 0.98	18.41 ± 1.05*	32.51 ± 1.35*	36.54 ± 0.81*	15.70 ± 0.56*	16.68 ± 1.70*	26.34 ± 1.14*	12.92 ± 0.82*	20.00 ± 0.71*	20.88 ± 0.69*
**HP-β-CD**												
**CD/DPPC 1:1**	1.36 ± 0.43	38.30 ± 0.67	41.45 ± 0.71	1.12 ± 0.37	14.78 ± 0.29	14.28 ± 1.14	0.98 ± 0.08	1.66 ± 0.16	5.77 ± 0.21	1.26 ± 0.19	1.64 ± 0.36	9.56 ± 0.39
**CD/DPPC 10:1**	6.86 ± 0.68*	39.40 ± 0.22	44.52 ± 0.63	5.36 ± 0.76*	15.17 ± 0.10	20.40 ± 0.89*	2.61 ± 0.37*	3.64 ± 0.29*	3.02 ± 0.87*	2.18 ± 0.30*	2.30 ± 0.25	2.40 ± 0.51
**CD/DPPC 100:1**	16.7 ± 0.48*	45.69 ± 2.39	49.35 ± 0.97	17.19 ± 1.04*	32.90 ± 0.76*	36.70 ± 0.24*	13.70 ± 0.50*	18.27 ± 0.27*	23.71 ± 0.91*	6.98 ± 0.25*	11.00 ± 0.64*	12.91 ± 1.65*
**SBE-β-CD**												

**CD/DPPC 1:1**	2.58 ± 0.29*	39.58 ± 0.15	42.52 ± 0.48	1.76 ± 0.10*	15.69 ± 0.58	14.08 ± 0.24	1.30 ± 0.45*	2.96 ± 0.42	6.27 ± 0.15	0.73 ± 0.18	1.33 ± 0.32	7.46 ± 1.16
**CD/DPPC 10:1**	7.11 ± 0.38*	41.52 ± 0.26	45.43 ± 0.41	5.94 ± 0.42*	16.27 ± 0.60	21.59 ± 1.12*	3.21 ± 0.75*	1.98 ± 0.71*	8.35 ± 0.27*	3.04 ± 0.44*	1.03 ± 0.08	3.71 ± 1.11
**CD/DPPC 100:1**	13.90 ± 0.53*	48.37 ± 1.73	52.70 ± 0.54	16.64 ± 1.62*	28.19 ± 1.06*	31.36 ± 0.74*	11.9 ± 0.18*	20.25 ± 0.70*	21.59 ± 0.80*	10.71 ± 0.07*	13.30 ± 0.87*	14.32 ± 0.21*

Table S4: The SRB release values obtained at time t_0, 4, and 24 h for γ-CD-treated liposomes at different CHOL content and different CD/DPPC molar ratios.

The SRB release value of blank liposomes was 0% at t_0 for the different formulations. Values are expressed as the means of three different measurements ± SD (* indicates a value of $p < 0.05$ statistically significant. The p values compare the membrane permeability in the presence of CD to that of the respective blank.)

	DPPC/CHOL (100:10)			DPPC/CHOL (100:25)			DPPC/CHOL (100:50)			DPPC/CHOL (100:100)		
**Time**	**0 h**	**4 h**	**24 h**	**0 h**	**4 h**	**24 h**	**0 h**	**4 h**	**24 h**	**0 h**	**4 h**	**24 h**
**Blank**	0	43.64 ± 1.84	48.34 ± 1.73	0	12.60 ± 1.28	15.01 ± 1.35	0	3.13 ± 0.79	4.17 ± 1.26	0	2.41 ± 0.70	5.15 ± 1.35
**γ-CD/DPPC 1/1**	0.60 ± 0.11	43.49 ± 0.47	49.66 ± 0.81	0.74 ± 0.30	14.81 ± 0.12	17.07 ± 0.29	0.15 ± 0.05	1.50 ± 0.10	3.25 ± 0.66	0.13 ± 0.08	1.00 ± 0.11	6.04 ± 0.13
**γ-CD/DPPC 10/1**	2.57 ± 0.54*	32.95 ± 0.88	40.04 ± 0.59	1.04 ± 0.81	9.69 ± 0.34	14.38 ± 0.70	0.8 ± 0.12	1.5 ± 0.65	3.26 ± 0.73	0.45 ± 0.23	0.39 ± 0.20	3.66 ± 0.90
**γ-CD/DPPC 100/1**	6.90 ± 1.56*	39.29 ± 1.03	40.20 ± 1.84	2.63 ± 1.01*	23.18 ± 0.89*	24.00 ± 0.81*	2.70 ± 0.53*	6.04 ± 0.64*	10.19 ± 0.26*	1.76 ± 0.76	3.80 ± 0.23*	6.01 ± 0.69*
